# The Involvement of Microglial Cells in Japanese Encephalitis Infections

**DOI:** 10.1155/2012/890586

**Published:** 2012-08-07

**Authors:** Thananya Thongtan, Chutima Thepparit, Duncan R. Smith

**Affiliations:** ^1^Department of Biochemistry, Faculty of Medicine, Chulalongkorn University, Bangkok 10330, Thailand; ^2^Molecular Pathology Laboratory, Institute of Molecular Biosciences, Mahidol University, Nakorn Pathom 73170, Thailand; ^3^Center for Emerging and Neglected Infectious Diseases, Mahidol University, Nakorn Pathom 73170, Thailand

## Abstract

Despite the availability of effective vaccines, Japanese encephalitis virus (JEV) infections remain a leading cause of encephalitis in many Asian countries. The virus is transmitted to humans by *Culex* mosquitoes, and, while the majority of human infections are asymptomatic, up to 30% of JE cases admitted to hospital die and 50% of the survivors suffer from neurological sequelae. Microglia are brain-resident macrophages that play key roles in both the innate and adaptive immune responses in the CNS and are thus of importance in determining the pathology of encephalitis as a result of JEV infection.

## 1. Introduction

Japanese encephalitis virus (JEV) is a member of the Japanese encephalitis (JE) serocomplex of the genus *Flavivirus*, family Flaviviridae. It was originally isolated in 1935 from the brain of a 19-year old who died of encephalitis in Tokyo, Japan, and JE is now considered one of the most important encephalitic diseases worldwide [1–4]. Despite the existence of effective vaccines [5] which has led to a reduction in the incidence of JE in countries which have active immunization programs, JE cases appear to be increasing throughout Southeast Asia, probably as a result of increases in population density, deforestation, and the expanding irrigation of agricultural areas [2].

The JEV virion consists of a single-stranded positive sense genomic RNA of approximately 11 Kb, three structural proteins (the capsid, envelope, and membrane proteins) as well as a significant amount of lipid [6]. The genomic RNA is 5′-capped, but lacks a 3′-polyadenylated tail and encodes for a single open reading frame flanked by noncoding 5′ and 3′ ends that encodes the three structural proteins (with the membrane protein being encoded as a precursor pre-membrane (prM) form) as well as seven nonstructural proteins (NS1, NS2A, NS2B, NS3, NS4A, NS4B, and NS5). 

JEV is found in South, East, and Southeast Asia as well as in northern Australia, and JE predominantly occurs in China, India, and the countries of Southeast Asia [2, 7]. There are currently believed to be four distinct genotypes of JEV, genotypes I to IV [8], although some studies support the existence of a fifth JEV genotype [9, 10] all of which are thought to have arisen from a common ancestor virus present in the Indonesian-Malaysian region [8]. While some genotypes are present in multiple countries (such as genotype III), others are present in only one country, such as genotype IV which is found only in Indonesia [8]. Conversely countries may experience the circulation of several genotypes, such as Indonesia where genotypes II, III, and IV circulate or only one such as India where only genotype III is found [8]. Mackenzie and colleagues [11] noted that genotypes I and III were found predominantly in temperate, epidemic areas while genotypes II and IV were principally associated with tropical, endemic areas. However, genotypic shift, with the replacement of one genotype by another has occurred recently in several countries [12–14]. Currently, JEV is considered hyperendemic in northern India and southern Nepal as well as in parts of central and southern India and more than 3 billion people are living in the current JE-endemic region [2, 11].

JE occurs predominantly in rural and suburban areas where rice culture and pig farming coexist [11]. The natural transmission cycle of JEV is an enzootic cycle with transmission being maintained between the main natural reservoir which is water birds, particularly ardeid wading such as* Ardeola grayii* (pond heron) and *Bubulcus ibis* (cattle egret), and mosquitoes [2, 11], particularly *Culex tritaeniorhynchus* which is the principal vector of JE transmission in India and Southeast Asian countries [15]. Vertical transmission of JEV has been reported for several mosquito species including *C. tritaeniorhynchus, C. pipiens, C. annulus, C. quinquefasciatus, Aedes albopictus, A. togoi, *and* Armigeres subalbatus *mosquitoes [2, 16]. 

Pigs are recognized as the most important amplifying reservoir with respect to human infections [2, 16]. In pigs JEV infection is primarily associated with reproductive defects including abortions, stillborn fetuses, and the death of newborn pigs [17]. Young infected animals may occasionally show signs of encephalitis, while older (nonpregnant) animals generally only experience a transient febrile illness. The level of viraemia in pigs is sufficient to pass the virus onto feeding mosquitoes [2]. 

Other animals including horses and donkeys can be symptomatically infected by JEV. While JEV-infected horses can show mild symptoms including fever and anorexia, they can also show more severe clinical syndromes characterized by either lethargy with fever spikes, neck rigidity, and falling or staggering or by hyperexcitability with high fever, disordered behavior, blindness, sweating collapse, and eventual death. Other animals such as cattle and water buffaloes can be infected with JEV, but do not generate high virus titers. Experimental studies have shown that bats and lizards can be infected by ingestion of infected mosquitoes in addition to the mosquito feeding route, and both produce viraemia levels sufficient to infect mosquitoes leading to the proposal that they are possible hosts for maintaining JEV over winter in the northern part of its range [16], although this remains to be supported by field studies. 

Humans become infected after the bite of an infected mosquito. The infection does not cause a viraemia of sufficient level to infect subsequently feeding mosquitoes, so in terms of JEV transmission, humans are a dead-end host, although the consequences for the individual can be significant. The majority of infections are believed to be asymptomatic, with an asymptomatic: symptomatic ratio of 25–1000 : 1 [11]. The likelihood of an infection being symptomatic as opposed to being asymptomatic seems to be associated with a number of host factors including age, general health, and genetic makeup, as well as possible viral factors including strain virulence, route of entry, and amount of virus inoculated [2]. When illness does result, it is believed to occur after a 5 to 15 day incubation period with children under the age of 15 being the majority of cases [11]. Symptoms start with the sudden and abrupt onset of fever with headache in mild cases, while more severe disease is characterized by nausea and vomiting, neck stiffness, stupor, disorientation, convulsions, and paralysis [2, 11]. Acute flaccid paralysis and movement disorders, especially Parkinsonian features, have also been reported [2, 18, 19]. Nearly three-quarters of symptomatic patients show manifestations of encephalitis, while some patients additionally present with meningitis [2, 3, 20]. While some patients make a rapid recovery, up to 30% of JE cases admitted to hospital die and 50% of the survivors suffer from permanent neurological sequelae and even those JE patients with apparently good recovery have evidence of neurological deficit [4, 21]. An estimated 30,000 to 50,000 cases of JE occur annually, with an estimated 10,000 to 15,000 deaths [2, 3, 20]; however, these figures may be significantly underreported [22].

Following JEV infection, anti-JEV IgM is normally found earlier in the cerebrospinal fluid (CSF) than in the serum [23] and anti-JEV-specific IgM has been reported in the CSF as early as the first day of presentation of the patient, while serum anti-JEV IgM may only be found from day 9 of presentation [23]. Studies have suggested that high levels of anti-JEV IgM antibodies in the CSF are correlated with a good outcome [24]. In JE patients, levels of TNF-*α* in the serum and CSF are significantly increased [25], and mortality rate is correlated with increased concentrations of TNF-*α* in serum and CSF of JE patients [24]. Moreover, levels of IFN-*α*, the proinflammatory cytokine IL-6, and the chemokine IL-8 were all higher in the CSF of the nonsurvivors than in those of the survivors [26].

## 2. Encephalitis and Involvement of the Brain in JE

Despite the relatively low occurrence of encephalitis in light of the larger number of infections (both symptomatic and asymptomatic), JEV infections are the leading cause of encephalitis in children in Southeast Asia, accounting for some 40% of all cases of encephalitis [27, 28]. The mechanism by which the virus enters the brain is not fully known, but immunohistochemical studies, which have shown viral antigens in the brain capillary endothelial cells and diffuse infection of the brain, suggest a blood-borne route [29] with the blood brain barrier (BBB) being crossed by either passive or active transfer across endothelial cells [2]. Electron microscopy has supported an endocytic mode of viral transportation across the BBB in mouse brain [30]. Previous injury or damage to the BBB may also serve to increase the likelihood of transfer across the BBB. 

More recently it was proposed that macrophages can harbor JEV and the virus may employ a “Trojan horse” mechanism to infiltrate into the CNS [31, 32]. Human monocytes are susceptible to JEV infection although the level of replication is low and, significantly, infected monocytes do not undergo apoptosis and can persist for more than three weeks in culture [33]. In this way, JEV may reside inside monocytes during transport across the BBB [31, 32], leading to the subsequent infection of nearby CNS cells as the infected monocyte releases newly synthesized virus particles. A similar mechanism has been proposed for penetration of the BBB by human immunodeficiency virus (HIV) in cases of HIV-encephalitis [34]. This so-called “Trojan horse” mechanism is also supported by the finding that macrophage-derived neutrophil chemotactic factor (MDF) produced in JEV-infected mice can cause an influx of neutrophils into the CNS, because of the breach of the vascular endothelium lining [35]. Liu and colleagues working with a mouse model system suggested that changes in the BBB permeability occurred as a direct consequence of JE infection, which resulted in deformation of tight junctions and subsequent dissociation of endothelial cells allowing the entry of either JEV virions or infected leukocytes to the cerebrum as the initial site of infection, with subsequent spread to the cerebellum [36]. 

After virus infection CD8+ T lymphocytes are amongst the first mononuclear cells to arrive at the infected tissue, followed by interaction with antigen-presenting cells. Consequently an increasing number of inflammatory cells, including macrophages/microglia and finally antibody-secreting plasma cells, are attracted to the site of virus infection [37]. Generally, defects in the recruitment of inflammatory cells to the infected areas allow the virus to spread in the CNS. In contrast, antibodies to JEV may help in restricting the viral load while in its incubation period in peripheral lymphatic tissues [38, 39].

At the molecular level, JEV infection leads to the upregulation of matrix metalloproteinase 9 (MMP-9) on endothelial cells [40], which causes brain damage in mice owing to the degradation of components of the basal lamina and disruption of BBB. It was also found that JEV-induced MMP-9 expression in rat brain astrocytes (one of the components of BBB, which are also infected by JEV and may help in the neuroinvasion of JEV [2]) is mediated through a reactive oxygen species/c-Src/PDGFR/PI3 K/Akt/MAPKs-dependent AP-1 signaling cascade [41]. However, direct JEV infection of astrocytes does not result in the release of neurotoxic mediators, and it is activated microglia that promote neuronal loss [32, 42]. JEV is a neurotropic virus, and it is believed that the principal targets of the virus in the brain are neuronal cells [43]. Evidence suggests that neuronal cells are productively infected by JEV with the concomitant induction of strong proinflammatory and antiviral responses [44] and the eventual induction of apoptosis [45, 46]. Neuronal death or damage can lead to the subsequent astroglial and microglial activation and release of further proinflammatory mediators [46]. 

## 3. Microglia and JEV Infection

Microglia play key roles in both innate and adaptive immune response in the CNS. Circulating microglia precursors derived from circulating precursor mesodermal hematopoietic cells enter into the developing brain, and there are three subtypes of microglia according to their location, namely, parenchymal, perivascular, and leptomeningial microglia [32]. Microglia are mostly in an amoeboid phagocytic shape in the perinatal brain and these amoeboid microglia are later transformed to ramified resting microglia. They remain in this form until there is a stimulus to activate their transformation into amoeboid phagocytic cells and microglia are closely related to peripheral macrophages regarding their origin and phagocytic activity [47]. Resident microglial cells in the mature brain can replicate, but they can also be replaced by circulating monocytes [48]. The distribution of microglial cells in the brain remains somewhat controversial, but it is generally agreed upon that the distribution is not uniform throughout the brain [49–52], and a difference in the topographic distribution of activated, resting, and phagocytic microglia has been observed in JEV infection [53]. Human autopsy studies have shown that the grey matter areas of the brain including the thalamus and hippocampus are the primary JEV affected brain regions [20] and in animal model systems these areas have been associated with significant increases in the number of activated and phagocytic microglia [53], suggesting a good accordance between the presence of activated microglia and significant neuronal damage. 

JEV-infected neuronal cells secrete elevated levels of a number of pro-inflammatory cytokines and chemokines including IL-12, TNF-*α*, MCP-1, and IL-6 [54] which are capable of directly activating microglia [53]. In response, microglial cells can subsequently produce a number of pro- and anti-inflammatory cytokines including IL-1*β* and IL-18 [55, 56], both of which can induce bystander neuronal cell death [56]. In this manner, microglial cells could accentuate the damage, without being more directly involved in the infection process. However, a number of studies have shown that microglial cells can be directly infected with JEV [53, 57–62], and this is supported by studies that have shown the occasional colocalization of JEV antigens with microglial specific markers [53]. Significantly; however, JEV-infected microglia have been shown to express elevated levels of a number pro-inflammatory mediators including IL-18, IL-1*β*, TNF-*α*, IL-6, and RANTES [42, 56], which can induce neuronal cell death. In this manner, microglial cells may contribute to increased neuronal cell death through both infective and noninfective pathways. 

In one study microglial cells have been proposed to act as a long-lasting reservoir of JEV [61] as a consequence of BV2 mouse microglial cells remaining productively infected for up to 16 weeks without significant alteration of morphology. The potential for long lasting reservoirs is supported by studies that have shown viral persistence in the human nervous system in approximately 5% of JEV-infected patients [63]. The persistence of JEV in microglial cells in JEV patients might be analogous to the reported persistence of Chikungunya virus (CHIKV) in macrophages in a nonhuman primate model system [64]. The mechanism by which JEV enters into microglia remains unknown, although a number of molecules have recently been implicated as acting as potential receptors for JEV in mediating entry of the virus to microglial cells including CD4 [62] which is expressed by perivascular microglia. Levels of CD4 antigen have been shown to be modulated on microglial cells during their differentiation and activation as well as during dysfunction of the BBB [65]. Intriguingly, CHIKV has recently been shown to infect macrophages by hiding in apoptotic blebs from cells dying as a consequence of CHIKV infection [66], and as microglia are phagocytic cells this method of entry remains a possibility in cases of JEV infection. In CHIKV infections, infection of macrophages through engulfment of CHIKV containing apoptotic blebs does not raise a proinflammatory response [66], which may be particularly significant in the establishment of long term infection. 

Microglial cells express Toll-like receptors (TLRs), the family of pattern recognition receptors (PRRs) that function as part of the innate immune system by recognizing structurally conserved pathogen molecules, collectively called pathogen-associated molecular patterns (PAMPs). JEV is encoded by a single-stranded RNA genome which can be detected by TLR7/8 while RIG-I, a cytoplasmic PRR, and TLR3 can detect dsRNA which is formed during the replication of JEV. RIG-I is essential for the induction of IRF-3 and IFN *α*/*β* activation in JEV-infected microglial cells [67]. More recently the PRR NOD-like receptor NLRP3 has been implicated in the JEV-mediated production of the pro-inflammatory cytokines IL-1*β* and IL-18 from activated microglial cells [58]. NLRP3 is part of the inflammasome which responds to cellular infection or stress leading to the activation of caspase-1 and the maturation of pro-inflammatory cytokines [68] and depletion of NLRP3 by siRNA resulted in reduced activation of caspase-1 and decreased levels of IL-1*β* and IL-18 in JEV-infected microglia [58]. As the cytokines IL-1*β* and IL-18 have previously been implicated in bystander neuronal cell death [56] the activation of this pathway may explain a significant part of the neuronal damage seen in severe cases of JEV infection.

Infection with JEV was further found to elicit extracellular glutamate accumulation from microglia [57]. Glutamate, an excitatory neurotransmitter, is a neurotoxic substance that interacts with NMDA (N-methyl-D-aspartate) receptors on neurons leading to Ca^2+^ influx and eventual cell death, and glutamate-mediated excitoneurotoxicity has been identified as an important mechanism for neuronal death during virus infection [57, 69]. Astrocytes can reuptake glutamate to get rid of excess extracellular glutamate, but in pathologic conditions, TNF-*α* has been shown to downregulate astrocyte-mediated glutamate transport GLT-1 [69–71]. Microglia can be induced by autocrine TNF-*α* stimulation to upregulate glutaminase leading to extensive microglial glutamate release [72]. It was recently reported that JEV-infected microglia cultures contain high concentrations of glutamate. This was not observed in the supernatants of JEV-infected neuron or astrocyte cell cultures. TNF-*α* released by microglia in JEV infection stimulate microglial glutamate release by upregulating glutaminase expression involving protein kinase C, CREB, and C/EBP-*β* signaling in an autocrine manner [57].

Although initiation of the immune response by microglial cells is primarily an important protective mechanism in the CNS, as seen with JEV infection, unrestrained inflammatory responses may result in irreparable brain damage [53]. Microglial cells also produce the cytokine IL-10, which is normally released with or after the secretion of pro-inflammatory cytokines to limit pro-inflammatory cytokine production and to modulate the degree of the inflammatory response [73]. However, although studies have shown that IL-10 is able to limit bystander neuronal cell death in JEV infection, the levels of IL-10 decrease following JEV infection and are inversely proportional to the increased levels of pro-inflammatory cytokines [74], suggesting that a significant brake on the inflammatory process in JEV infection has been removed.

## 4. Conclusions and Future Directions

While neurons are believed to be the primary target of JEV in the brain, the evidence suggests that activated microglial cells are the main pro-inflammatory mechanism leading to a greatly increased level of neuronal damage. This is particularly damaging as microglia are the main neuroprotective mechanism, tasked with protecting neurons by dealing with infections [49]. The question arises therefore why the neuronal defense mechanism results in apparently increased neuronal damage. The evidence would suggest that microglia become activated both indirectly as a consequence of neuronal damage and directly as a result of infection of the microglia themselves. In this case microglia may serve to increase neuronal cell death through multiple mechanisms, all of which drive the production of pro-inflammatory cytokines, while downregulating cytokines that could serve to dampen the inflammatory response. Several recent studies have investigated a number of compounds that can interfere with the inflammatory process including minocycline [31, 40, 59, 75, 76], arctigenin [77], and rosmarinic acid [78], some of which may both increase neuroprotection directly as well as down regulate the pro-inflammatory response. The BBB itself represents a significant hurdle in developing compounds to treat the CNS inflammatory response in JE, and the majority of drugs for other conditions in current use capable of crossing the BBB are small lipid-soluble molecules that cross by transmembrane diffusion [79]. However, new approaches to drug delivery across the BBB include applications of nanotechnology [80], harnessing BBB transporter receptors such as the low-density lipoprotein-receptor-related protein-1 (LRP-1) through the use of peptide conjugates [81] and the use of liposomes [82] all of which may be capable of being adapted to deliver drugs to treat JEV-induced neuroinflammation. 

There is a single report in the literature describing JEV latency and recurrence of JE in children many months after the initial infection [83]. While this does not seem to be a common feature of JEV infection, evidence suggests that monocytes can remain infected long after apparent viraemia has stopped [33], and in this case further research is needed to determine whether the “Trojan horse” mechanism described earlier [31, 32] contributes to the neurological deficit over a significantly longer period of time than is currently assumed by the constant introduction of new virus into the CNS. 

In the short term, the incidence of JE may be responsive to improved mosquito control programs, coupled with improved animal husbandry methods. However studies have shown that arbitrarily increasing the distance between humans and pigs does not eliminate the risk of JEV to humans and that control needs to be placed in the context of the feeding and flight habits of particular mosquito species [84]. In the long term, complete control of JEV infection in humans will come through the development of safe, cheap vaccines that can be distributed widely to at risk populations. In this regard, the replacement of mouse-brain-produced JEV vaccines with cell-culture-produced vaccines is a significant step in the right direction [5], and the development of vaccines such as IMOJEV (previously called ChimeriVax-JE) which produces protective antibody levels in 95% (children) or over (adults) of recipients one month after a single dose administration [85] will move the focus from development to distribution.
